# The Effects of *Lilium lancifolium* Thunb. on the Alleviation of Joint Pain: A Randomized, Double-Blind, Placebo-Controlled Clinical Trial

**DOI:** 10.3390/life14091136

**Published:** 2024-09-09

**Authors:** Soomin Jeon, Hayera Lee, Jae-Ho Lee, Kippeum Lee, Dongki Hong, Soo-Dong Park, Jae-Jung Shim, Jung-Lyoul Lee, Jaehwan Lee, Jong-Cheon Joo

**Affiliations:** 1 R&BD Center, hy Co., Ltd., 22, Giheungdanji-ro 24beon-gil, Giheung-gu, Yongin-si 17086, Gyeonggi-do, Republic of Korea; 10003273@hy.co.kr (S.J.); yera@hy.co.kr (H.L.); jhlee@hy.co.kr (J.-H.L.); 10002903@hy.co.kr (K.L.); dkhong@hy.co.kr (D.H.); soodpark@hy.co.kr (S.-D.P.); jjshim@hy.co.kr (J.-J.S.); jlleesk@hy.co.kr (J.-L.L.); 2 Department of Sasang Constitutional Medicine, College of Korean Medicine, Wonkwang University; Iksan 54538, Jeonbuk, Republic of Korea

**Keywords:** *Lilium lancifolium*, osteoarthritis, joint pain, quality of life, health functional food

## Abstract

Arthritis is mainly a geriatric disease that causes joint pain and lowers the quality of life. This clinical trial was performed to evaluate the efficacy of *Lilium lancifolium* Thunb. (HY-LL) in alleviating joint pain. Six candidate anti-inflammatory components including regaloside A were identified in HY-LL using HPLC analysis. All participants were assigned to the HY-LL or the placebo group and took tablets twice a day for 12 weeks. As a result, pain VAS and K-WOMAC total scores significantly decreased after 12 weeks compared to the baseline in the HY-LL group, with a statistically significant difference between the two groups (*p* = 0.043, 0.043). The K-WOMAC sub-scores for pain and function showed a statistically significant improvement in the HY-LL group compared to the placebo group (*p* = 0.023, 0.047). Furthermore, the participants’ overall quality of life improved after 12 weeks of HY-LL consumption (*p* = 0.024). However, no significant differences were observed in the blood biomarkers. Therefore, this study demonstrated the positive effect of 12 weeks of HY-LL consumption on joint pain and quality of life.

## 1. Introduction

In modern society, the increasing elderly population is accompanied by an increase in aging-associated diseases, such as arthritis and hypertension. Arthritis is one of the main geriatric diseases that lowers the quality of life [1,2,3]. It causes joint pain, reduces the ability to exercise, and limits daily life activity. Degenerative arthritis, also called osteoarthritis, is an inflammatory disease that causes joint pain, stiffness, and edema mainly due to degenerative damage to the cartilage [4]. Generally, aging is the primary cause of the disease but other factors can also contribute such as gender, genetic factors, obesity, and lifestyle habits [5,6]. The goals of arthritis treatment are to minimize pain, prevent joint destruction, reduce long-term joint deformities and disabilities, and use medications that are less burdensome for the patient. Arthritis treatment is primarily surgical, encompassing procedures such as curettage arthroplasty, artificial joint replacement, and synovectomy [7,8,9]. While surgery offers a quick recovery, it carries risks such as infection. Pharmacological treatments include intra-articular steroid or hyaluronic acid injections, oral non-steroidal anti-inflammatory drugs, and analgesics [10]. These medications provide only temporary relief and can cause side effects, including hypersensitivity reactions, immune system deterioration, cognitive impairment, and digestive and cardiovascular disorders. Consequently, the importance of alternative medicines, such as safe and effective functional foods, is increasing.

*Lilium lancifolium* Thunb. is a perennial flower of the Liliaceae family, found in Korea, Japan, China, and Sakhalin [11]. The flowers are colorful and widely cultivated for ornamental purposes, while the bulbs are used for food and medicinal purposes. Since ancient times, the bulb of *L. lancifolium* has been used in traditional medicine for its therapeutic properties in treating bronchitis, pneumonia, laryngitis, severe stress, and diabetes [12,13,14]. It contains alkaloids, starch, proteins, fats, and pharmacological active components such as *p*-coumaric and sinapic acid [15]. Recent studies have demonstrated its potential in alleviating arthritis symptoms and inhibiting inflammation through cell culture and animal models [16,17]. However, clinical studies on the effectiveness of *L. lancifolium* in alleviating arthritis symptoms in humans have still not been reported.

This study was designed to investigate the effects of *L. lancifolium* extract on joint health in humans. This research was guided by our in vivo and in vitro experiments, which demonstrated anti-inflammatory and cartilage-protective effects through the reduction of inflammatory cytokines [18]. Accordingly, this study aimed to scientifically evaluate the effect of *L. lancifolium* on joint health by conducting a human clinical trial.

## 2. Materials and Methods

### 2.1. Component Analysis Using High-Performance Liquid Chromatography

All trial materials were provided by hy Co., Ltd. (Yongin, Republic of Korea). The extract powder was prepared as follows: Dried *Lilium lancifolium* (LL) bulbs were extracted using 50% ethanol at a ratio of 5 to 10 times the weight of the bulbs. The mixture was stirred at 60 ± 5 °C for 18 to 24 h. The resulting extract was concentrated, and maltodextrin, equivalent to 50% of the solid content, was added. Finally, the mixture was lyophilized to obtain the extract powder.

The components of LL were analyzed using two different high-performance liquid chromatography (HPLC) methods—Method A and Method B.

Method A—High-performance liquid chromatography (HPLC) analysis for determining regaloside A in *L. lancifolium* extract was performed following a previously described method with modification [19]. Chromatographic separation was performed in a Shiseido Capcell Pak UG120 C-18 column (Shiseido, Japan; 4.6 mm × 250 mm, 5 μm) with a gradient method using mobile phases composed of 0.1% formic acid in distilled water (A) and 90% acetonitrile (B).

Method B—The simultaneous analysis of various phenolic compounds, including syringic acid, caffeic acid, coumaric acid, ferulic acid, and hesperetin, in *L. lancifolium* was performed using HPLC as follows: The sample extract was filtered through a 0.45 μm RC membrane, and ten μL of the solution was injected into the HPLC 1260 Infinity system (Agilent, Waldbronn, Germany). Chromatographic separation was performed on a ZORBAX SB-C18 column (Agilent, Santa Clara, CA, USA; 4.6 mm × 250 mm, 5 μm) with a gradient method using mobile phases composed of 0.1% formic acid in distilled water (A) and 0.1% formic acid in acetonitrile (B). The gradient elution was performed as follows: 0–2 min, 0%, B; 2–45 min, 0–50%, B; 45–55.1 min, 50–95%, B; 55.1–70 min, 0%, B. The column temperature was maintained at 40 °C, and the flow rate was 0.8 mL/min. The detection wavelength was 280 nm. Phenolic compounds were identified by comparing the chromatograms with those of commercial standards.

### 2.2. Ethics and Study Design

This study, conducted at Wonkwang University Korean Medicine Hospital (Jeonju, Jeollabuk-do, Republic of Korea) from 2021 to 2022, was designed as a 12 week, randomized, double-blind, placebo-controlled clinical trial with two parallel groups receiving either *L. lancifolium* (HY-LL) or placebo tablets. The institution’s Independent Institutional Review Board reviewed and approved the research protocol and subject consent form (IRB no. WUJKMH-IRB-2021-0005). The clinical trial adhered to the ethical principles outlined in the Korean Clinical Research Information Service Helsinki and the Korean Declaration of Good Clinical Practice.

A screening test was conducted only on volunteers who provided written consent, and research subjects meeting the selection criteria were selected. Study subjects revisited the hospital within 2 weeks of their screening visit and were enrolled after meeting the inclusion criteria. They consumed HY-LL or a placebo twice a day for 12 weeks and were assigned to the corresponding groups. At the second visit (baseline), participants were randomly allocated to receive either the experimental treatment or a placebo. A statistician used a computer-generated list to determine the random assignments. Both participants and investigators remained blinded to the treatment allocation until the study’s conclusion. During the intake period, patients visited the Wonkwang University Korean Medical Hospital every 6 weeks to evaluate efficacy, vital signs, drug administration history, changes in their medical condition, and adverse reactions.

### 2.3. Subjects

All subjects were informed of the study purpose and protocol, as well as the foreseeable risks associated with the trial. The inclusion criteria were as follows: (1) adults aged 50–80 years, (2) individuals experiencing pain for more than 3 months with pain Visual Analog Scale (VAS) scores of 30–70 mm, (3) those with a Kellgren–Lawrence (KL) grade of 1–2, and (4) those who voluntarily decided to participate and signed the agreement to adhere to the study precautions.

The exclusion criteria were as follows: (1) receiving an intra-articular steroid injection within the past 3 months; (2) receiving an intra-articular hyaluronic acid injection within the past 1 month; (3) receiving an intra-articular joint fluid injection within the past 6 months; (4) positive rheumatoid factor or suspected rheumatoid arthritis; (5) a BMI < 18.5 kg/m^2^ or >35 kg/m^2^; (6) a history of knee surgery; (7) a history of lower extremity fracture or radiating pain due to spinal joint disease within the past 3 months; (8) nervous system or lower extremity joint abnormalities that could impede walking, in addition to knee joint discomfort due to aging; (9) diseases that might affect joints, such as gout and fibromyalgia; (10) acute or chronic cardiovascular, musculoskeletal, or gastrointestinal diseases; (11) use of medications or functional foods for joint health; (12) receiving antipsychotic medications; (13) alcoholism, drug abuse, or suspected alcoholism or drug abuse; (14) participation in other clinical trials within the past 3 months; (15) diagnostic test results showing AST or ALT > 3 times reference ranges, serum creatinine > 2.0 mg/dL, and creatine kinase > twice the reference range; (16) pregnant or lactating women; (17) women of childbearing age not using appropriate contraceptive methods; and (18) exclusion by the principal investigator of the study due to the results of diagnostic tests or other reasons.

### 2.4. Investigational Products

The research subjects consumed the same product every day for 12 weeks. Both groups took four tablets orally each day, with two tablets in the morning and two in the evening. Each tablet weighed 400 mg, and the tablets in the HY-LL group contained 250 mg of HY-LL per tablet. Therefore, participants ingested a total of 1.6 g of tablets per day (4 tablets in total), with the test group consuming 1 g of HY-LL daily. Both placebo and experimental tablets looked identical to the naked eye.

### 2.5. Measurements

In addition to the investigational product (IP), the following 20 items were measured to assess the differences between the HY-LL and placebo groups and to evaluate the suitability of the test: name, gender, age, occupation, living environment, drinking status and amount, smoking period, amount of smoking, medical and medication histories, physical examination (including interview, examination, auscultation, percussion, and palpation), sitting blood pressure, pulse rate, height and weight, and chest X-ray. Additionally, the Global Physical Activity Questionnaire (GPAQ) was administered to assess usual activity levels and lifestyle habits [20].

#### 2.5.1. KL Grade

The Kellgren–Lawrence (KL) grading system is used to evaluate osteoarthritis through knee X-ray examination [21]. Grade 0 indicates a normal joint. Grade 1 suggests doubtful joint space narrowing with possible osteophyte formation. Grade 2 denotes the presence of osteophytes and possible joint space narrowing. Grade 3 is characterized by definite joint space narrowing, severe multiple osteophytes, bone sclerosis, and possible bone deformation. Finally, Grade 4 is marked by a significantly narrowed joint space, large osteophytes, severe osteosclerosis, and definite bone deformities.

#### 2.5.2. Pain Visual Analog Scale

The degree of pain due to osteoarthritis was assessed using the pain Visual Analog Scale (VAS), where subjects marked their pain level on a 100 mm line. A higher score on this scale indicates more severe pain.

#### 2.5.3. Korean Western Ontario and McMaster University Osteoarthritis Index

The Korean Western Ontario and McMaster University Osteoarthritis Index (K-WOMAC) is a Korean version of WOMAC, which is a survey tool for assessing arthritis and joint diseases [22]. It consists of 24 questions related to knee joint pain, categorized into three subcategories—pain, stiffness, and function (Table S1). Higher scores in each subcategory indicate more severe symptoms and greater limitations in daily activity.

#### 2.5.4. WHOQOL-BREF (WHO Quality of Life—BREF)

The World Health Organization Quality of Life—BREF (WHOQOL-BREF) is a condensed version of the WHOQOL questionnaire, which is designed to measure quality of life (Table S2) [23]. It consists of 26 items divided into 6 subcategories—overall quality of life, general health, physical health, psychological health, social relationships, and environmental quality of life. Responses to each item are recorded on a 5-point Likert scale and are then converted to a 100-point scale using the conversion table provided by the WHO. For negatively worded items (items 3, 4, and 26), scores were adjusted by subtracting each domain score from 6.

### 2.6. Compliance and Safety Evaluation

Blood cytokines and inflammatory indicators were measured. The cytokines assessed included interleukin-6 (IL-6) and tumor necrosis factor-alpha (TNF-α). The inflammatory indicators measured were prostaglandin E2 (PGE-2), cyclooxygenase-2 (COX-2), high-sensitivity C-reactive protein (hs-CRP), and erythrocyte sedimentation rate (ESR). Hematological tests (white and red blood cell counts), biochemical tests (alkaline phosphatase and aspartate aminotransferase), urinalysis, and pregnancy tests were conducted to evaluate the safety of HY-LL. Additionally, to determine whether there were differences in blood factors based on usual sitting time, inflammatory indicators and cytokines were compared in subjects whose sitting time exceeded 500 min, as reported in the Global Physical Activity Questionnaire (GPAQ).

Investigational products were returned at the second and third visits, and compliance was assessed based on the remaining amount. Compliance was calculated as the ratio of the number of products consumed compared to the number of products required during the test period. The safety of the food supplements was evaluated by monitoring adverse events (AEs), hematological tests, biochemical tests, urinalysis, and vital signs.

### 2.7. Statistical Analysis

Statistical analysis was performed using the SAS program (Version 9.4; SAS Institute, Cary, NC, USA). The protocol data set (PP set) was used to evaluate the effectiveness of HY-LL. The Chi-square test or Fisher’s exact test and independent *t*-tests were performed to determine the difference in baseline distribution between the groups.

After 12 weeks of treatment, comparisons between the groups were made using the independent *t*-test, while comparisons within groups were conducted with the paired *t*-test. Analysis of covariance (ANCOVA) was performed, adjusting for baseline values on non-homogeneous efficacy evaluation items and demographic information items as covariates. All data were presented as mean ± standard deviation (SD). Statistical significance was determined using a two-tailed test with a significance level set at *p* < 0.05.

## 3. Results

### 3.1. Components Included in HY-LL

To identify the candidate functional compounds in HY-LL, high-performance liquid chromatography (HPLC) was performed (Table 1). As a result, regaloside A was identified as the main component of HY-LL using Method A. Additional HPLC analysis was performed using Method B to further understand the phenolic compounds in HY-LL; five phenolic compounds were identified—caffeic acid, syringic acid, *p*-coumaric acid, ferulic acid, and hesperetin.

### 3.2. The Characteristics of Study Subjects

**Figure 1:**
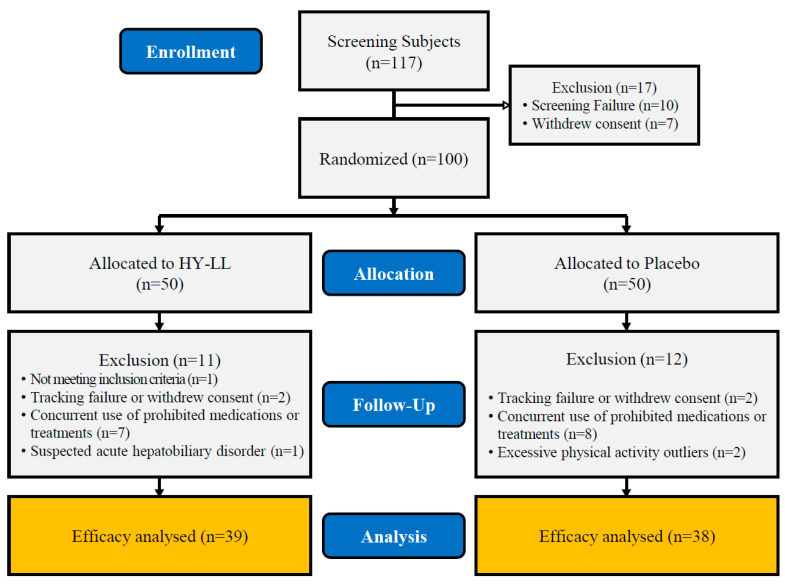
Flow diagram showing the selection and allocation of participants in the study.

After excluding 17 participants, 100 subjects were randomly assigned and participated in this clinical study (Figure 1 and Table 2). Although we attempted to recruit an equal ratio of men to women, it was difficult to find men who met the inclusion and exclusion criteria. The average height of the participants was 159.75 ± 6.96 cm, with the HY-LL group measuring 159.38 ± 5.97 cm and the placebo group measuring 160.12 ± 7.87 cm. The average weight was 61.95 ± 8.26 kg, with the HY-LL group averaging 62.75 ± 8.98 kg and the placebo group averaging 61.14 kg ± 7.47 kg. No statistically significant differences were found in general characteristics, except for career type. Additionally, no significant differences were found in blood pressure, alcohol consumption, physical activity, and dietary intake.

Biomedical analysis was conducted to assess the randomization and safety of this study (Table 3). No statistically significant differences were observed in hematological parameters. In the biochemical test, urinalysis pH decreased by −0.04 ± 1.19 (*p* = 0.813) in the HY-LL group and increased by 0.50 ± 1.14 (*p* = 0.003) in the placebo group, indicating a statistically significant difference between the groups (*p* = 0.023). However, these changes were within the normal range, and no clinical significance was identified. Therefore, no safety issues were recorded.

### 3.3. Results of Symptom Questionnaire Related to Joints

**Figure 2:**
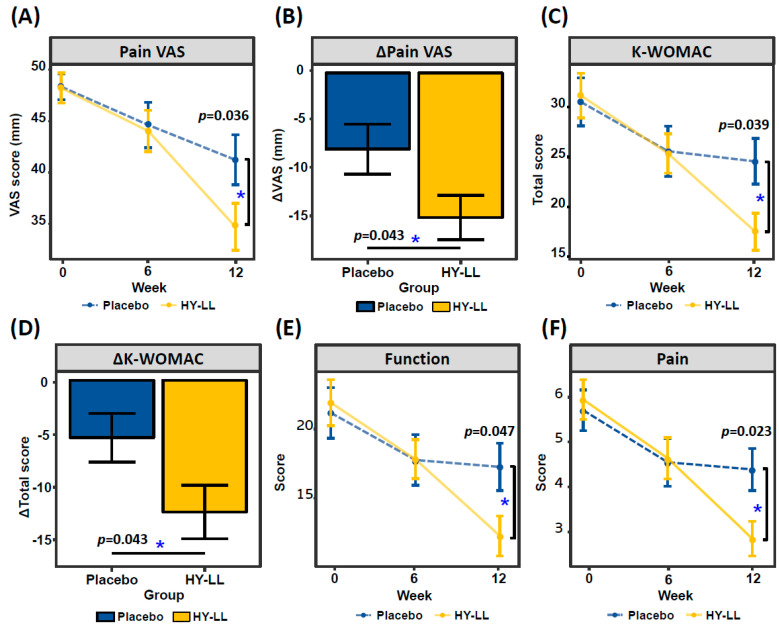
Effects of HY-LL and placebo on joint pain. (**A**) Pain VAS score by visiting term; (**B**) the changing value of pain VAS over 12 weeks; (**C**) the K−WOMAC score by visiting term; (**D**) the changing value of K−WOMAC over 12 weeks; (**E**) the sub-score of K−WOMAC relating to joint function; (**F**) the sub-score of K-WOMAC related to joint pain. The statistical difference is shown by *, indicating *p* < 0.05.

Subjects’ joint health and pain were evaluated to determine the effect of the oral intake of HY-LL on the human body (Figure 2). After 12 weeks, both groups exhibited a statistically significant decrease in the pain VAS, with a greater reduction observed in the HY-LL group. The comparison of changes over the 12-week period between the two groups revealed a significant difference (*p* = 0.043).

After 12 weeks, the K-WOMAC score significantly decreased to −13.67 ± 17.21 in the HY-LL group and −5.97 ± 15.51 in the placebo group compared to baseline. There was a significant difference between the two groups regarding the change from baseline. Specifically, the K-WOMAC sub-scores showed significant differences in pain and function between the two groups following the 12-week intake. The scores decreased to −3.10 ± 3.22 for pain and −9.67 ± 13.42 for function in the HY-LL group, compared to −1.32 ± 3.55 and −3.92 ± 11.38 in the placebo group, respectively. These differences were statistically significant (*p* = 0.023 for pain and *p* = 0.047 for function).

### 3.4. Effect of HY-LL on Quality of Life According to WHOQOL-BREF

**Figure 3:**
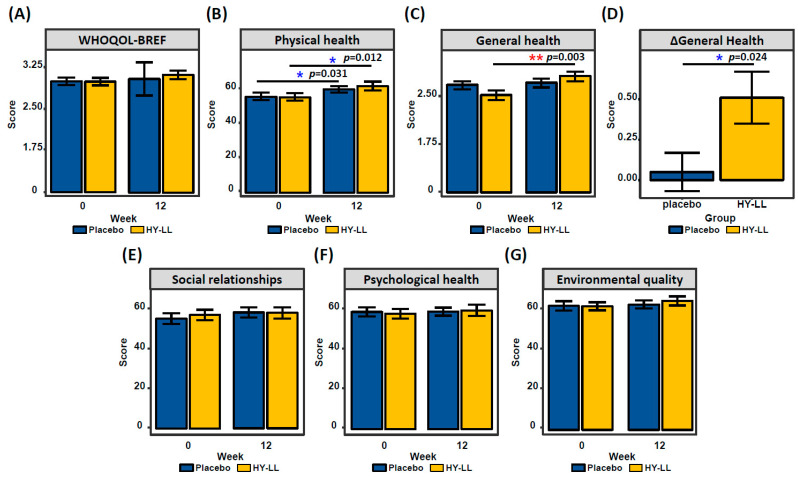
Change in WHOQOL-BREF categories depending on intake period. (**A**) Overall quality of life score, (**B**) physical health score, (**C**) general health score, (**D**) changing value of general health, (**E**) social relationships score, (**F**) psychological health score, and (**G**) environmental quality score for 12 weeks. Statistical difference is shown by * and **, indicating *p* < 0.05 and *p* < 0.01, respectively.

The WHOQOL-BREF measured the improvement in participants’ quality of life due to HY-LL consumption (Figure 3). After 12 weeks of treatment, the general health score significantly increased to 0.51 ± 1.00 points in the HY-LL group and 0.05 ± 0.73 points in the placebo group, with a statistically significant difference between the groups (*p* = 0.024). Although there was no statistically significant difference, the HY-LL group exhibited a higher average increase in overall quality of life, general health, physical health, and environmental quality of life after 12 weeks of intake compared to the placebo group.

### 3.5. Effect of HY-LL on Hematological Factors

**Figure 4:**
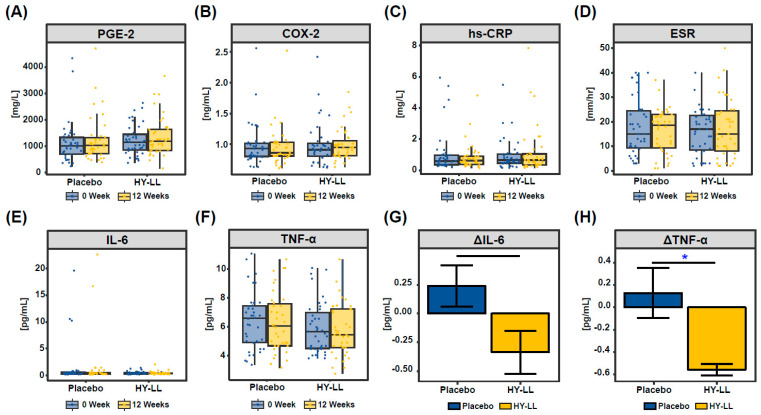
Change in inflammatory cytokines depending on intake period. (**A**) PGE-2, (**B**) COX-2, (**C**) hs-CRP, (**D**) ESR, (**E**) IL-6, and (**F**) TNF-α concentration in blood for 0 and 12 weeks in all groups. Comparison of change values of (**G**) IL-6 and (**H**) TNF-α concentration in the blood between the placebo and HY-LL groups. Statistical difference is shown by *, indicating *p* < 0.05.

The blood collected from the participants was analyzed to measure inflammatory indicators and cytokines, and to assess the effect of HY-LL on joint pain relief and inflammation (Figure 4). No significant differences were observed between the HY-LL and placebo groups before or after the 12-week period, and no changes were noted with prolonged intake. However, participants with prolonged sedentary lifestyles (>500 min) exhibited a notable reduction in IL-6 and TNF-α levels depending on the duration of HY-LL intake, compared to the placebo group (*p* = 0.057 and *p* = 0.036, respectively). No significant differences were found between the groups for the other four indices—PGE-2, COX-2, hs-CRP, and ESR.

## 4. Discussion

Arthritis in the elderly is a condition that causes pain and limitations in daily life, significantly impacting quality of life. This study aimed to confirm the joint pain relief effects of HY-LL through a clinical trial. After 12 weeks of consumption, improvements were observed in participants’ joint pain, daily discomfort, and inflammatory biomarkers. Since previous studies have linked plant extract components to biological mechanisms, we analyzed the components of HY-LL to identify potential functional agents. Our analysis detected five types of polyphenols and regaloside A in HY-LL. These components are well regarded for their anti-inflammatory effects (Figure 1). Regaloside A, a phenolic acid glyceride with anti-inflammatory properties, has been experimentally shown to improve cell morphology and inhibit inflammatory cytokines [24,25]. Syringic acid and *p*-coumaric acid have been reported to alleviate arthritis by inhibiting Il-6 inflammation and osteoclast formation, respectively, in a rat arthritis model [17,26,27]. Additionally, ferulic acid, another HY-LL component, has been found to mitigate osteoarthritis chondrocyte toxicity by inhibiting the production of IL-6, PGE-2, and sub-MMP-9 and activating the SIRT1/AMPK/PGC-1α signaling pathway [28]. Given the association of these functional components with inflammatory responses, we hypothesized that HY-LL might reduce inflammation. Our inflammatory biomarker analysis supports this hypothesis. Although not statistically significant, the HY-LL group showed a greater average reduction in IL-6 and TNF-α compared to the placebo (Figure 4). These findings suggest that the pain relief experienced by the subjects may be linked to a reduction in inflammatory reaction. IL-6 and TNF-α are well known to be involved in rheumatoid arthritis, with IL-6 promoting joint inflammation and destruction through cell signaling [29], and TNF-α initiating an inflammatory response leading to joint swelling and bone destruction [30,31]. The observed decrease in IL-6 and TNF-α in the HY-LL group, compared to the placebo group, indicates a potential reduction in inflammatory response. Therefore, it is likely that HY-LL intake decreased joint inflammation biomarkers, leading to reduced inflammation and pain relief.

This reduction in inflammation may have alleviated arthritis-related pain. After 12 weeks of consumption, joint pain was assessed to have improved in the HY-LL group, as indicated by pain VAS and K-WOMAC scores (Figure 2). Pain VAS scores provide a direct measurement of pain, clearly demonstrating that joint pain decreased following HY-LL intake. The K-WOMAC scale, which assesses joint pain and function in detail, also showed significant improvements with HY-LL consumption. This scale evaluates daily joint pain and difficulties associated with activities such as climbing stairs and sleeping, highlighting that HY-LL intake led to notable improvements in both pain and functional aspects. Furthermore, the WHOQOL-BREF results showed a higher overall increase in quality of life scores for the HY-LL group. Specifically, the general health score was significantly higher, with improvements also noted in psychological health and environmental quality of life after 12 weeks of HY-LL intake. This suggests that HY-LL not only alleviated joint pain, but also contributed to a broader enhancement in daily life quality (Figure 3). Our findings are consistent with previous studies on functional foods and joint pain improvement. For instance, one study demonstrated that collagen hydrolysate consumption for six months alleviated pain in patients with primary knee osteoarthritis, as measured by the VAS and K-WOMAC pain sub-score [32]. Similarly, another study found that joint health improved through the use of FlexPro MD^®^ (a combination of krill oil, astaxanthin, and hyaluronic acid) with significant results in pain VAS and K-WOMAC pain subcategory scores [33]. Unlike these previous studies, our research shows significant improvements across multiple measures, including pain VAS, K-WOMAC, and WHOQOL-BREF. This comprehensive approach enhances the robustness of our findings and contributes to a more effective understanding of joint health improvements through HY-LL.

In our previous research, the anti-inflammatory effects of HY-LL were demonstrated through reductions in inflammatory markers, including TNF-α, IL-6, and CRP, in both mouse and beagle models [18]. These findings suggested that the components within HY-LL could be beneficial in alleviating joint pain and enhancing quality of life.

Building on these results, our current study aimed to investigate the impact of 12 weeks of HY-LL consumption on arthritis pain and overall life improvement. We assessed pain relief using pain VAS scores and the K-WOMAC, and evaluated quality of life with the WHOQOL-BREF. Our findings indicate that HY-LL not only alleviated joint pain, but also improved participants’ quality of life. We anticipate that these results will support the development and industrialization of functional foods derived from natural products for arthritis pain relief.

## 5. Conclusions

In this clinical trial, the effects of HY-LL on arthritis pain and quality of life were evaluated over a 12-week period. HY-LL demonstrated a significant efficacy in alleviating joint pain, as measured by the Visual Analog Scale (VAS) and the Korean Western Ontario and McMaster University Osteoarthritis Index (K-WOMAC). Given that arthritis not only causes pain but also diminishes overall quality of life, this study aims to offer valuable insights into the potential of functional foods for alleviating arthritis-related pain.

## Figures and Tables

**Table 1:** Simultaneous determination of polyphenolic compounds in HY-LL using HPLC.

Analysis Method	Compound	Retention Time (min)	Concentration (μg/g) ^a^
Method A	Regaloside A	12.765	341.43 ± 8.07
Method B	Caffeic acid	20.843	26.34 ± 0.05
	*p*-coumaric acid	24.728	16.04 ± 0.14
	Ferulic acid	26.323	27.23 ± 2.02
	Hesperetin	39.551	24.19 ± 0.20

^a^ All values are presented as average ± standard deviation.

**Table 2:** Baseline data of study participants. All values are presented as average ± standard deviation.

	HY-LL (*n* = 39)	Placebo (*n* = 38)	*p*-Value
Sex (M/F)	5/34	5/33	>0.99
Age (years) ^a^	58.33 ± 5.05	60.24 ± 6.61	0.161
Height (cm)	160.00 ± 5.94	158.76 ± 7.64	0.431
Weight (kg)	63.91 ± 9.11	60.39 ± 7.86	0.074
BMI (kg/m^2^) ^b^	24.99 ± 3.56	23.97 ± 2.80	0.165
SBP (mmHg) ^c^	119.54 ± 12.27	120.82 ± 10.77	0.629
DBP (mmHg) ^d^	73.36 ± 10.07	73.68 ± 9.11	0.882

^a^ All values are presented as average ± standard deviation. ^b^ BMI is abbreviation of Body Mass Index. ^c,d^ SBP and DBP is abbreviation of Systolic Blood Pressure and Diastolic Blood Pressure.

**Table 3:** Measurement of biochemical parameters.

Variable	HY-LL (*n* = 39)			Placebo (*n* = 38)			*p*-Value
	Baseline	12 Week	Change Value	Baseline	12 Week	Change Value	
WBC ^a^	5.84 ± 1.26	5.79 ± 1.33	−0.05 ± 1.08	5.69 ± 1.18	5.73 ± 1.39	0.04 ± 1.23	0.710
RBC ^b^	4.49 ± 0.39	4.57 ± 0.40	0.07 ± 0.19	4.52 ± 0.31	4.55 ± 0.29	0.03 ± 0.15	0.231
Hemoglobin	13.64 ± 1.05	13.80 ± 1.15	0.16 ± 0.55	13.87 ± 0.99	13.86 ± 0.97	−0.01 ± 0.50	0.107
ALP ^c^	195.56 ± 59.84	198.08 ± 56.16	2.52 ± 34.47	201.64 ± 54.95	202.52 ± 49.60	0.88 ± 27.09	0.792
AST ^d^	25.64 ± 10.01	25.94 ± 8.23	0.30 ± 11.13	25.14 ± 6.39	25.96 ± 8.60	0.82 ± 6.16	0.773
ALT ^e^	24.78 ± 14.09	26.70 ± 16.84	1.92 ± 18.01	23.44 ± 10.16	24.28 ± 12.15	0.84 ± 11.19	0.720
Albumin	4.28 ± 0.23	4.27 ± 0.28	−0.01 ± 0.30	4.30 ± 0.22	4.25 ± 0.30	−0.05 ± 0.27	0.531
BUN ^f^	14.39 ± 3.90	13.88 ± 2.70	−0.51 ± 3.28	14.15 ± 3.37	14.09 ± 4.03	−0.06 ± 4.22	0.553
Creatinine	0.83 ± 0.13	0.81 ± 0.13	−0.02 ± 0.10	0.85 ± 0.18	0.83 ± 0.18	−0.02 ± 0.11	0.775
Glucose	97.20 ± 10.11	97.26 ± 12.28	0.06 ± 9.59	97.12 ± 12.25	97.28 ± 12.19	0.16 ± 8.03	0.955
Urine pH	5.91 ± 1.01	5.87 ± 1.01	−0.04 ± 1.19	5.61 ± 0.74	6.11 ± 1.04	0.50 ± 1.14	0.023 *

All values are presented as average ± standard deviation. Statistical significance is shown by *. ^a–f^ WBC(White Blood Cell), RBC(Red Blood Cell), ALP(Alkaline Phosphatase), AST(Aspartate Transaminase), ALT(Alanine Transaminase), and BUN(Blood Urea Nitrogen).

## Data Availability

The data presented in this study are available in the article and Supplementary Materials. The raw data are available upon reasonable request from the corresponding author.

## Supplementary Materials

The following supporting information can be downloaded at: https://www.mdpi.com/article/10.3390/life14091136/s1, Table S1. K-WOMAC scale; Table S2: WHOQOL-BREF.
