# Supplementary material for: The Effects of Lilium lancifolium Thunb. on the Alleviation of Joint Pain: A Randomized, Double-Blind, Placebo-Controlled Clinical Trial
Source: Life (Basel). 2024 Sep 9;14(9):1136. doi: 10.3390/life14091136 (PMC11432755; doi:10.3390/life14091136)
Supplement: Supplementary file 1 [file life-14-01136-s001.zip › life-3137775-supplementary.pdf]

Table S1. K-WOMAC scale

Pain [Score: 0-20]					
Think about the pain you have felt in your knee or hip joints in the last 48 hours because of your arthritis. (Please mark "V" for the appropriate answer)					
	None(0)	Slightly (1)	Average (2)	Severe (3)	Very Severe (4)
1. When walking on flat ground					
2. When walking up and down the stairs					
3. Disturbed by pain that interrupts sleep when sleeping at night					
4. When sitting (On a chair) or when lying down					
5. When standing upright					
**Stiffness [Score: 0~8]**					
Think about the stiffness you have been feeling in your joints due to arthritis in the last 48 hours. Stiffness means that when you move your joints, they don't move as well as they normally would and they feel heavy.					
	None(0)	Slightly (1)	Average (2)	Severe (3)	Very Severe (4)
1. How sever is the joint stiffness when you wake up in the morning?					
2. How severe is the stiffness you feel after sitting (on a chair), lying down, or resting in the afternoon?					
**Function [Score: 0~68]**					
Think about how your arthritis has affected your physical functioning over the past 48 hours. Physical functioning refers to your ability to get around and take care of your body (please mark the appropriate answer with a "V").					
	None(0)	Slightly (1)	Average (2)	Severe (3)	Very Severe (4)
1. When going down the stairs					
2. When going up the stairs					

3. When sitting (On a chair) and standing up					
4. When standing					
5. When bending over towards the floor					
6. When walking on flat ground					
7. When boarding or getting off the passenger vehicle or the bus					
8. When going grocery shopping					
9. When putting on socks or stockings					
10. When rising from bed					
11. When taking off socks or stockings					
12. When going to bed					
13. When going in and out of the bathtub					
14. When sitting (on a chair)					
15. When sitting on or standing up from the toilet					
16. When performing physically stressful labor					
17. When performing light physical labor					

Table S2. WHOQOL-BREF

This questionnaire asks how you feel about your health, economic, social and mental quality of life over the past two weeks. Choose the one answer that seems most appropriate.						
Question		Not at all (1)	A little (2)	A moderate amount (3)	Very much (4)	An extreme amount (5)
1	How would you rate your quality of life?					
2	How satisfied are you with your health?					

The following questions ask about how much you have experienced certain things in the last two weeks.						
3	To what extent do you feel that (physical) pain prevents you from doing what you need to do?					
4	How much do you need any medical treatment to function in your daily life?					
5	How much do you enjoy life?					
6	To what extent do you feel your life to be meaningful?					
7	How well are you able to concentrate?					
8	How safe do you feel in your daily life?					
9	How healthy is your physical environment?					
The following questions ask about how completely you experience or were able to do certain things in the last two weeks.						
10	Do you have enough energy for everyday life?					
11	Are you able to accept your bodily appearance?					
12	Have you enough money to meet your needs?					
13	How available to you is the information					

	that you need in your day-to-day life?					
14	To what extent do you have the opportunity for leisure activities?					
15	How well are you able to get around?					
The following questions ask you to say how good or satisfied you have felt about various aspects of your life over the last two weeks.						
16	How satisfied are you with your sleep?					
17	How satisfied are you with your ability to perform your daily living activities?					
18	How satisfied are you with your capacity for work?					
19	How satisfied are you with yourself?					
20	How satisfied are you with your personal relationships?					
21	How satisfied are you with your sex life?					
22	How satisfied are you with the support you get from your friends?					
23	How satisfied are you with the conditions of your living place?					
24	How satisfied are you with your access to health services?					

25	How satisfied are you with your transport?					
The following question refers to how often you have felt or experienced certain things in the last two weeks.						
26	How often do you have negative feelings such as blue mood, despair, anxiety, depression?					
